# A Mild Causal Relationship Between Tea Consumption and Obesity in General Population: A Two-Sample Mendelian Randomization Study

**DOI:** 10.3389/fgene.2022.795049

**Published:** 2022-02-24

**Authors:** Cancan Li, Mingyun Niu, Zheng Guo, Pengcheng Liu, Yulu Zheng, Di Liu, Song Yang, Wei Wang, Yuanmin Li, Haifeng Hou

**Affiliations:** ^1^ School of Public Health, Shandong First Medical University and Shandong Academy of Medical Sciences, Taian, China; ^2^ Beijing Key Laboratory of Clinical Epidemiology, School of Public Health, Capital Medical University, Beijing, China; ^3^ The Second Affiliated Hospital of Shandong First Medical University, Taian, China; ^4^ Centre for Precision Health, School of Medical and Health Sciences, Edith Cowan University, Perth, WA, Australia; ^5^ Department of Endocrinology, Taian City Central Hospital, Taian, China

**Keywords:** tea consumption, obesity, mendelian randomization analysis, causal association, single nucleotide polymorphism

## Abstract

Evidence from observational studies for the effect of tea consumption on obesity is inconclusive. This study aimed to verify the causal association between tea consumption and obesity through a two-sample Mendelian randomization (MR) analysis in general population-based datasets. The genetic instruments, single nucleotide polymorphisms (SNPs) associated with tea consumption habits, were obtained from genome-wide association studies (GWAS): UK Biobank, Nurses’ Health Study, Health Professionals Follow-up Study, and Women’s Genome Health Study. The effect of the genetic instruments on obesity was analyzed using the UK Biobank dataset (among ∼500,000 participants). The causal relationship between tea consumption and obesity was analyzed by five methods of MR analyses: inverse variance weighted (IVW) method, MR-Egger regression method, weighted median estimator (WME), weighted mode, and simple mode. Ninety-one SNPs were identified as genetic instruments in our study. A mild causation was found by IVW (odds ratio [OR] = 0.998, 95% confidence interval [CI] = 0.996 to 1.000, *p* = 0.049]), which is commonly used in two-sample MR analysis, indicating that tea consumption has a statistically significant but medically weak effect on obesity control. However, the other four approaches did not show significance. Since there was no heterogeneity and pleiotropy in this study, the IVW approach has the priority of recommendation. Further studies are needed to clarify the effects of tea consumption on obesity-related health problems in detail.

## Introduction

Obesity is a nutrition-related metabolic disorder caused by genetic and environmental determinants (González-Muniesa et al., 2017; Blüher, 2019). Obesity and obesity-related diseases have been becoming major public health burdens worldwide. In the United States, the healthcare expense was about $1,901 per year for each obese person, which extrapolated to about $149.4 billion at the national level (Kim and Basu, 2016). Due to the continuous rise of incidence in the past 50 years, obesity has now reached pandemic proportion (Blüher, 2019; Chooi et al., 2019), and is predicted to be 20% by 2025 ((NCD Risk Factor Collaboration (NCD-RisC), 2016). Moreover, obesity increases the risk of various diseases, such as type 2 diabetes, cardiovascular disease, dementia and cancers (Blüher, 2019). In spite of the crucial role of diet and exercise in the treatment of obesity, supportive herbal remedies are of increasing concern (Liang et al., 2019).

Tea is one of the popular beverages globally, which is consumed up to 2 billion cups per day (Drew, 2019). Tea is considered an anti-obesity beverage attributed to three main components: tea polyphenols, tea polysaccharides, and caffeine (Wang et al., 2014; Xu et al., 2015; Chen et al., 2018b). Although growing researches have focused on the relationship between tea and anti-obesity, the findings are inconsistent (Jurgens et al., 2012; Baladia et al., 2014; Li X. et al., 2020; Lin et al., 2020).

Conventional epidemiological studies are susceptible to the potential confounders and inverse causality, which over-or under-estimate the causal relationship between determinants and outcomes. Mendelian randomization (MR) analysis is able to control the biases by introducing instrumental variables (Cao et al., 2019). In MR studies, genetic variants that are closely associated with exposure factors are defined as instrumental variables, by which the causations between exposures and disease outcomes are measured by genetic variants as substitution (Zhang et al., 2020). Since the formation of gametes follows the Mendelian law of “parental alleles randomly assigned to offspring”, genetic variation is not affected by traditional confounding factors and is associated with outcomes in a time-sequential manner (Emdin et al., 2017). In the current study, a two-sample MR analysis was used to assess the causal relationship between tea consumption and obesity in general population-based databases.

## Materials and Methods

### Datasets

Obesity is a chronic disorder featured by excessive adiposity and defined by body mass index (BMI) ≥ 30 kg/m^2^ (ICD10: E66) for adults (WHO, 2004). For the datasets of exposure, significant single nucleotide polymorphisms (SNPs) related to tea consumption (*p* < 5 × 10^–6^) were obtained from a genome-wide association study (GWAS) among ∼120,000 participants of European ancestry, which included participants from the UK Biobank, Nurses’ Health Study, Health Professionals Follow-up Study and Women’s Genome Health Study (Supplementary Table S1). The summary statistics associated with tea consumption from the largest GWAS consortium were used to control the bias of participant overlap as well as to increase the statistical power (Wang et al., 2020; Zhang et al., 2021b). Exposure data were collected using a 24 h recall questionnaire (Oxford WebQ), where tea consumption refers to drinking any type of tea without other substances (*e.g.,* sugary). (Zhong et al., 2019). The linkage disequilibrium (LD) of significant SNPs linked to tea consumption was set to meet *r* ^2^ < 0.001 to avoid the effect of strong LD on the results. The outcome datasets of obesity were obtained from the UK Biobank study which recruited about 500,000 European participants aged 37–73 years from 2006 to 2010. The relevant data were extracted from two datasets respectively, including SNP sites, alleles, effect estimates for exposure and outcome (BETA), standard error (SE), and *p* values.

### Statistical Analysis

There are three premises for two-sample MR (Emdin et al., 2017; Greenland, 2018): 1) Genetic variation as an instrumental variable must be closely related to exposure. 2) Instrumental variables are not associated with any known confounders. 3) The instrumental variables are not directly related to the outcome, that is, the instrumental variables cannot affect the outcome in other ways except through the exposure factors (Supplementary Figure S1).

Prior to two-sample MR analysis, there is a need to unify the effect-value directions of exposure data and outcome data. Exposure and outcome data are unified into a dataset by removing the intermediate allele frequencies of SNPs containing palindromes (Hemani et al., 2018). In addition, SNPs with A/T or G/C alleles are defined as palindromic SNPs, “intermediate allele frequencies” referred to 0.01 < allele frequency < 0.30 (Hartwig et al., 2016).

Inverse variance weighted (IVW) method, MR-Egger regression method, weighted median estimator (WME), weighted mode, and simple mode were used to evaluate the causal effect between tea consumption and obesity, and subsequently checked the stability and reliability of the results. The IVW model is a weighted linear regression model, which is based on the premise that all genetic variants are valid instrumental variables (Yuan et al., 2020). MR-Egger regression method can obtain unbiased estimation when there is pleiotropy in instrumental variables, measure average pleiotropy through intercept term, and perform sensitivity analysis (Bowden et al., 2015). WME can still calculate the causal association effect when the genetic variation below 50% violates the core assumptions of MR (Bowden et al., 2016). The weighted mode is effective when the majority of instrumental variables are valid, even though other instrumental variables in the method do not meet the requirements of MR causal inference (Hartwig et al., 2017). The simple mode is a model-based estimation method that provides robustness for pleiotropy, although it is not as powerful as IVW (Milne et al., 2017).

Mendelian Randomization Pleiotropy Residual Sum and Outlier (MR-PRESSO) global test was used to appraise the pleiotropy and to identify the outliers (Verbanck et al., 2018). The intercept of the MR-Egger regression line illustrated the magnitude of the genetic pleiotropy. It was considered that there was no pleiotropic effect if no significant difference between intercept and 0 (*p* > 0.05) (Bowden et al., 2015). Cochran’s Q statistic and *I* ^*2*^ statistic were used to assess the heterogeneity among the estimates from included SNPs. The funnel plot showed the relationship between the individual Wald ratio of each SNP and its accuracy, and whether its symmetry indicated whether the results had directional horizontal pleiotropy (Choi et al., 2020). The “leave-one-out” method was used for sensitivity analysis. By gradually eliminating each SNP and calculating the combined effect of the remaining SNPs, the influence of individual SNP on results and the stability of the results were evaluated (Mokry et al., 2016). With regard to the overlap between the participants from which these summary statistics were generated and the outcome dataset, the analyses of Bias and Type 1 Error Rate for Mendelian Randomization with Sample Overlap were carried out to assess the potential bias caused by population overlap (https://sb452.shinyapps.io/overlap/) (Burgess et al., 2016). Finally, F statistics (Eq. (1)) were used to test the strength of the association between the SNPs as instrumental variables and tea consumption, and an *F* statistic >10 indicates a lower risk of weak instrumental variable bias (Zhang Q. et al., 2021; Chen et al., 2021).

$$F\;\mathrm{statistic}=\frac{R^{2}\times \left(N-2\right)}{\left(1-R^{2}\right)}$$ (1)

$$R^{2}=2\times \mathrm{eaf}\times \left(1-\mathrm{eaf}\right)\times \mathrm{Bet}a^{2}$$ (2)

N represents the sample size; eaf represents effect allele frequency.

All data analyses were performed by the “TwoSampleMR” package in R version 4.0.2 (R Foundation for Statistical Computing, Vienna, Austria). A power calculation was conducted using mRnd (https://cnsgenomics.com/shiny/mRnd/) (Brion et al., 2013). Statistical significance was set as two-tailed *p* < 0.05 unless otherwise specified.

## Results

### Instrumental Variable Selection

A total of 108 significant SNPs (*p* < 5 × 10^–6^, LD *r* ^2^ < 0.001) were obtained from the GWAS about tea consumption (Zhong et al., 2019). Among them, 16 SNPs were removed for being palindromic with intermediate allele frequencies, and one SNP was removed because of no corresponding outcome data. Finally, 91 SNPs were selected to perform the following MR analysis.

The detailed information of these SNPs was shown in Supplementary Table S2, mainly including effect allele (EA), other allele (OA) and summary statistics (beta coefficient, SE, and *p*-value).

### MR Analysis

**FIGURE 1:**
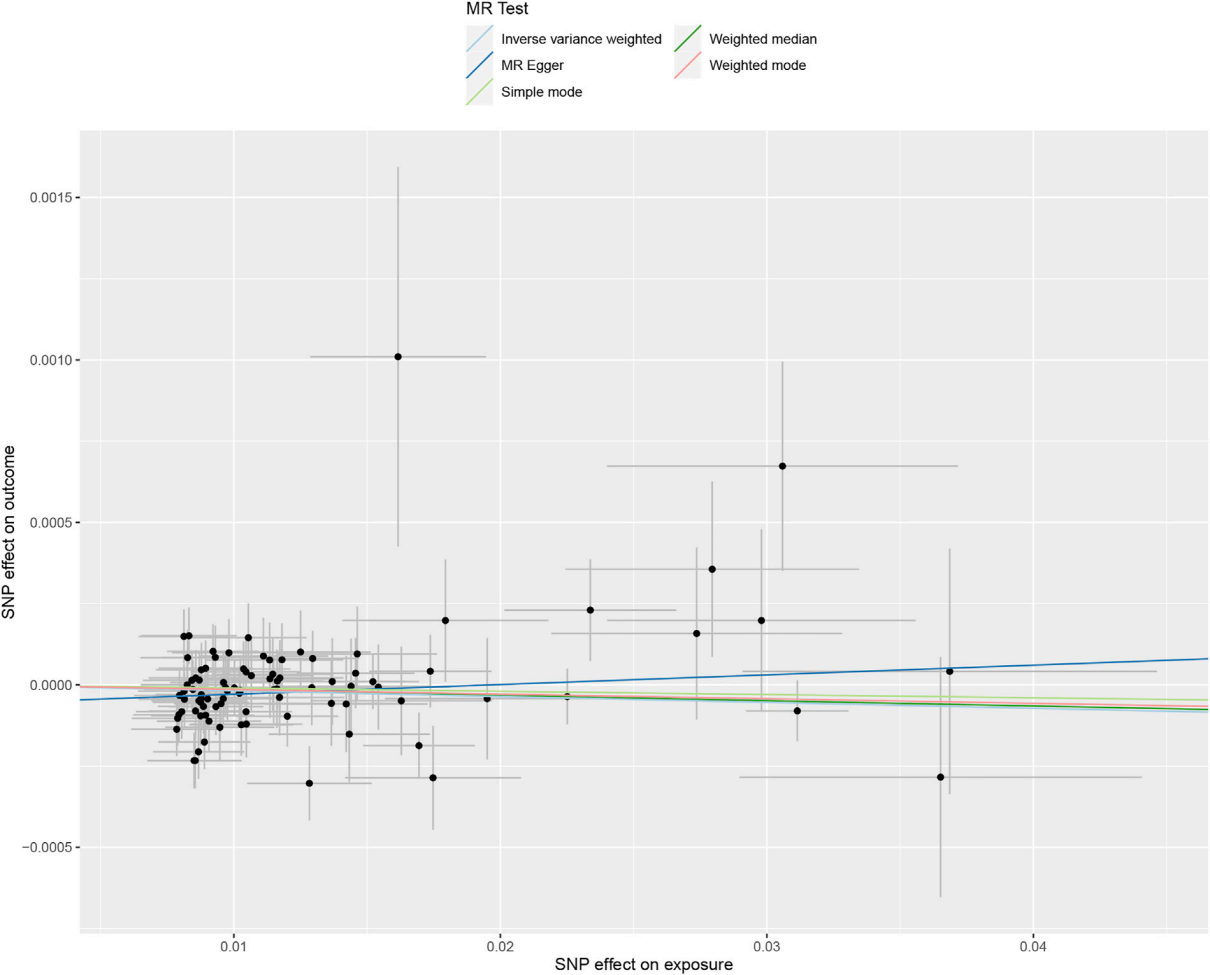
Scatter plot to visualize the causal effect between tea consumption and obesity. The slope of the straight line indicates the magnitude of the causal association, scatter plot of inverse variance weighted (IVW) method, MR-Egger regression method, weighted median estimator (WME), weighted mode and simple mode. MR, Mendelian randomization; SNP, single nucleotide polymorphism.

**FIGURE 2:**
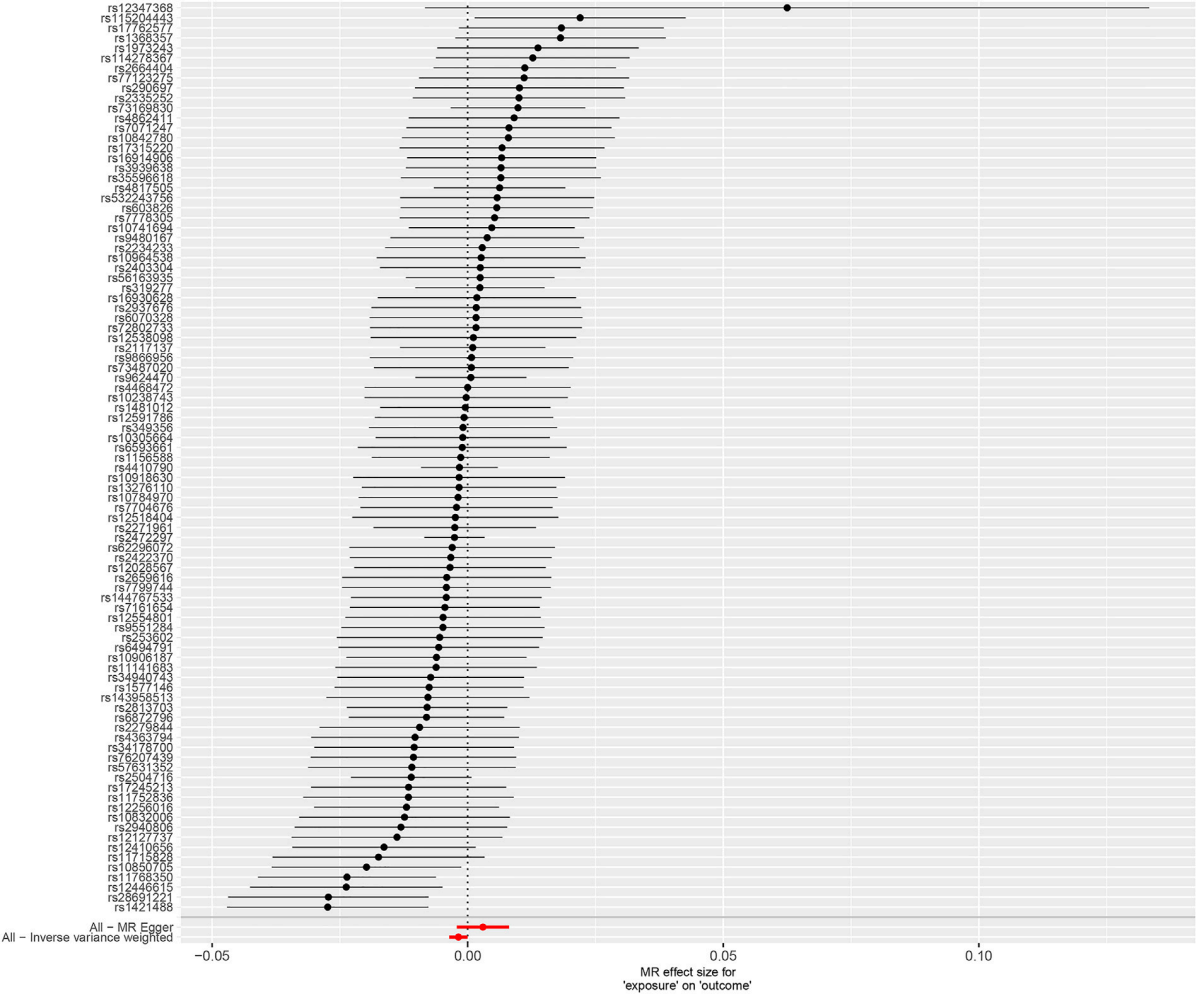
Forest plot to show the causal effect of tea consumption on obesity. Forest plot of IVW and MR-Egger regression method. MR, Mendelian randomization.

The causation between tea consumption and obesity was analyzed using the methods of IVW, MR Egger, WME, weighted mode, and simple mode, independently. As shown in Table 1 and Figure 1, a statistical significance was observed in IVW method analysis [odds ratio (OR) = 0.998, 95% confidence interval (CI) = 0.996 to 1.000, *p* = 0.049] (Table 1; Figures 1, 2). No significant causal relationships were observed in the analyses of MR Egger (OR = 1.003, 95% CI = 0.998 to 1.008, *p* = 0.255), WME (OR = 0.998, 95% CI = 0.996 to 1.001, *p* = 0.262), weighted mode (OR = 0.999, 95% CI = 0.994 to 1.003, *p* = 0.505) or simple mode (OR = 0.999, 95% CI = 0.993 to 1.005, *p* = 0.747) (Table 1; Figures 1, 2). In addition, a high statistical power (>85%) was identified in our study.

**TABLE 1:** Two-sample Mendelian Randomization for tea consumption on obesity risk.

Method	N SNPs	Beta coefficient	SE	OR (95%CI)	*p*
IVW	91	−0.002	0.001	0.998 (0.996–1.000)	0.049
MR-Egger	91	0.003	0.003	1.003 (0.998–1.008)	0.255
WME	91	-0.002	0.001	0.998 (0.996–1.001)	0.262
Weighted mode	91	-0.001	0.002	0.999 (0.994–1.003)	0.505
Simple mode	91	-0.001	0.003	0.999 (0.993–1.005)	0.747

N SNPs, the number of single nucleotide polymorphisms; SE, standard error; OR, odds ratio; CI, confidence interval; IVW, inverse variance weighted; WME, weighted median estimator.

### Sensitivity Analysis

**FIGURE 3:**
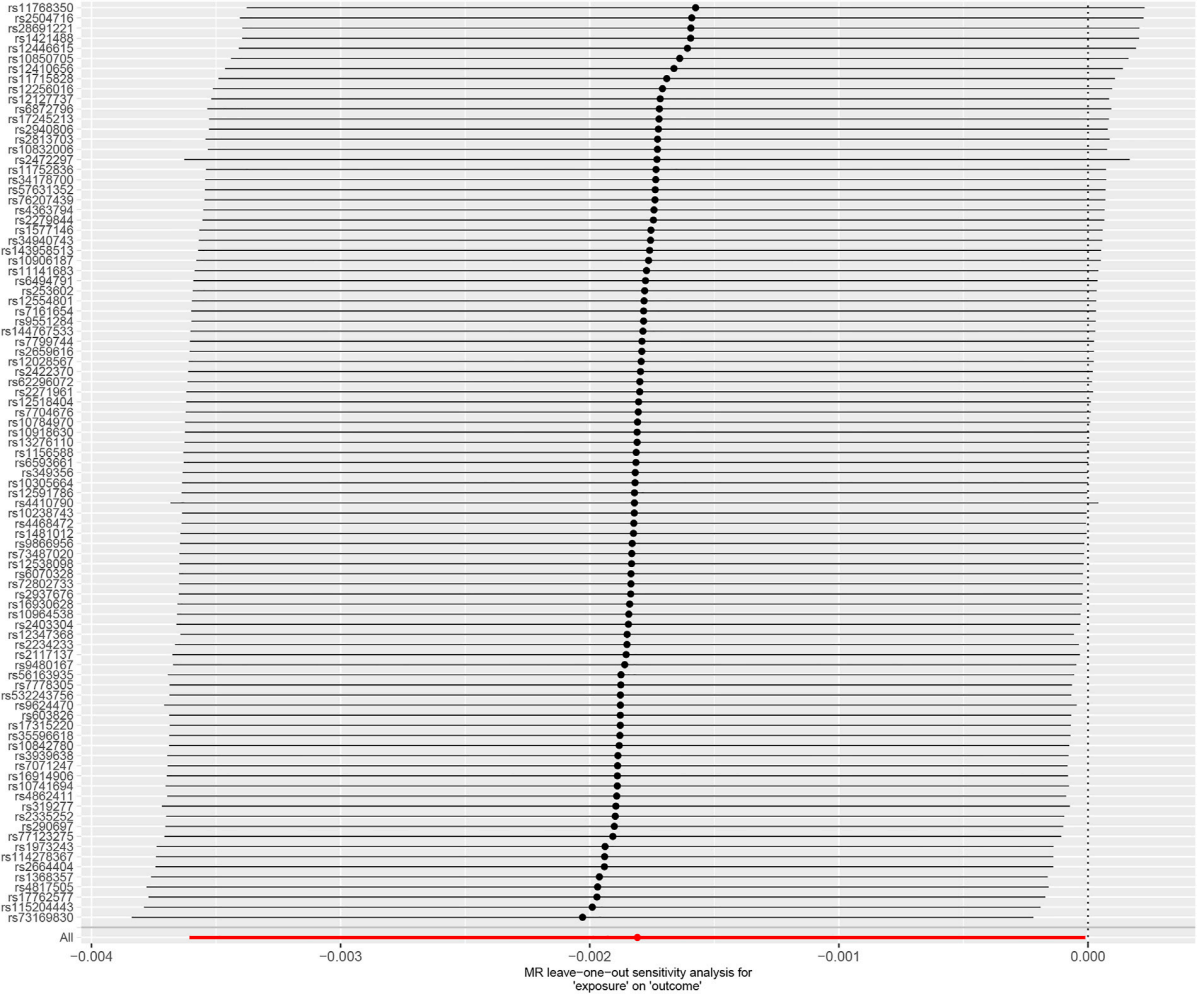
Forest plot of “leave-one-out” sensitivity analysis method to show the influence of individual SNP on the results. MR, Mendelian randomization.

**FIGURE 4:**
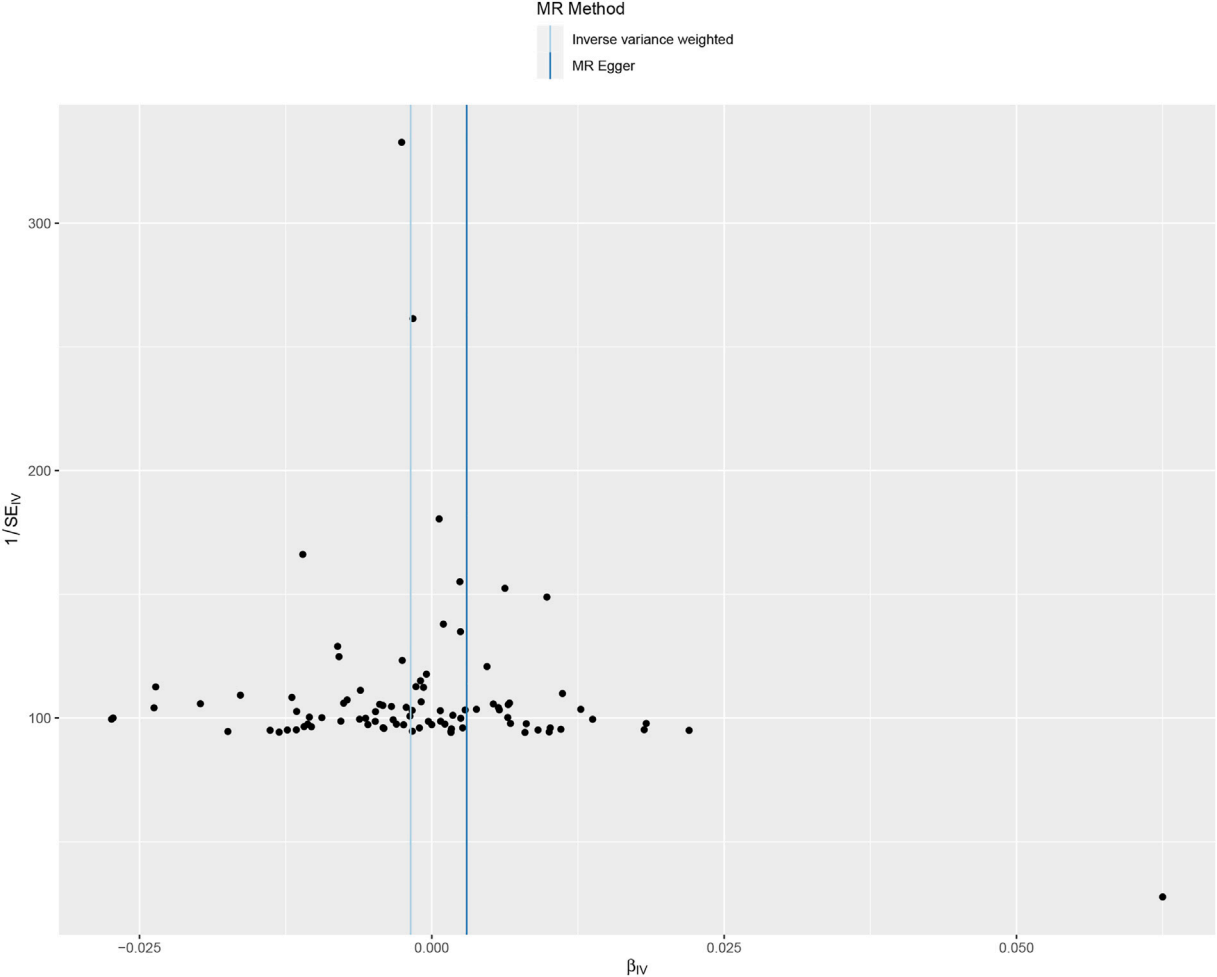
Funnel plot to visualize overall heterogeneity of Mendelian randomization assessment for the effect of tea consumption on obesity. MR, Mendelian randomization; SE, standard error; IV, instrument variable.

The leave-one-out method displayed that the results of the current two-sample MR analysis were strong (Figure 3), indicating that no instrumental variables influenced the causal inference. Our Cochrane Q-test showed no significant heterogeneity across the estimates of included SNPs (*p* = 0.464, *I* ^*2*^ = 0.61%). The funnel plot analysis illustrated a symmetry result (Figure 4), by which non-significance in directional and horizontal multipolarity was observed. And the genetic pleiotropy was not identified by the MR-Egger regression analysis (*p* = 0.053) or MR-PRESSO global test (*p* = 0.654), indicating that our findings were not influenced by the polymorphisms. As for population overlap, the overlap percentage was 17.17% (85852/500000). In addition, the bias and Type 1 error rate with sample overlap were <0.001 and 0.05 respectively, demonstrating that our results were less likely affected by sample overlap bias. Finally, no significant instrumental variable bias was observed by the F statistics (F > 10, ranging from 11.23 to 140.36).

## Discussion

Our two-sample MR analyses are conducted in five independent approaches. The result of IVW analysis evidenced that individuals drinking tea might have a 0.2% decrease in risk for obesity compared to those who do not. This mild causal relationship between tea consumption and obesity indicated that tea consumption has a statistically significant but medically weak effect on obesity control. The results of other approaches, including MR Egger, WME, weighted mode, and simple mode analyses, did not show causation between tea consumption and obesity. Since there was no heterogeneity and pleiotropy in this study, IVW results had the priority of recommendation. Besides, IVW is the most widely used and usually provides predominant results (Dan et al., 2020; Yuan et al., 2020).

It should be concerned whether instrumental variables in this study are associated with potential confounders. Firstly, the included SNPs were reported in the GWAS using statistical models on 24 h recall data, which were adjusted for age, sex, BMI, and top 20 principal components of population sub-structure (Zhong et al., 2019). Secondly, the GWAS studies for potential confounders (*i.e.*, energy intake and expenditure, exercise, physical activity, and sleep duration) have been examined, consequently, there were no overlap SNPs between tea consumption and potential confounders (De Moor et al., 2009; Doherty et al., 2018; Jiang et al., 2018; Williams et al., 2021). Although there might be other confounder-associated SNPs that have not been reported in these studies, we did not observe a significant association between potential confounders and the instrumental variables selected in our study.

As a popular, economical, and safe drink, the effect of tea on obesity is widely understood, especially among overweight and obese individuals (Wang et al., 2014; Ahmad et al., 2015; Xu et al., 2015; Chen et al., 2018b). The potential mechanisms of tea on obesity are as follows: 1) reducing food intake and energy absorption (Yang et al., 2016), 2) regulating the expression of lipid metabolism genes and inhibiting fat accumulation (Chen et al., 2017), 3) enhancing the activity of antioxidant defense enzymes (Ren et al., 2015; Chen et al., 2018a), 4) regulating intestinal microflora disturbance and attenuating intestinal inflammation (Li Y. et al., 2020; Zhou et al., 2020), and 5) maintaining intestinal barrier integrity (Lu et al., 2019).

White adipose tissue (WAT) is one of the body’s adipose tissues, and its browning can increase the body’s energy expenditure. The consumption of tea could induce the browning of WAT through the activation of the AMP-activated protein kinase (AMPK) signaling pathway via the upregulation of uncoupling protein-1 (UCP-1) expression (Yamashita et al., 2014; Wu et al., 2018). In addition, tea can reduce fat synthesis by inhibiting fat synthases [fatty acid synthase (FAS) and stearoyl-CoA desaturase (SCD)] and fat synthesis transcription factor [sterol regulatory element-binding protein-1c (SrebP-1C)] (Li et al., 2016; Sun et al., 2019).

In recent years, a growing number of studies have begun to explore the association between tea consumption and obesity. For example, one RCT study found that the intake of black Chinese tea extract (BTE) (333 mg/day) before meals for 12 weeks induced a decrease in both BMI and weight (Kubota et al., 2011). Another study found that drinking 8 g of oolong tea per day could decrease body fat content (He et al., 2009). In addition, one cohort study published in 2021 [relative risk (RR) = 0.767, 95% CI = 0.738 to 0.796, *p* < 0.05] and two meta-analyses published in 2020 came up with the positive results of the effect of tea on obesity (Li X. et al., 2020; Lin et al., 2020; Zhang et al., 2021c). Our study, a two-sample MR analysis based on general population-based datasets, verified the causal relationship between tea consumption and obesity.

Traditional epidemiological studies, consisting of case-control studies and cohort studies, provide representative findings on the relationship between exposures and outcomes. However, these studies are usually biased by confounding factors and adverse causal effects (He et al., 2009; Vernarelli and Lambert, 2013; Cai et al., 2021). MR analysis can control the biases by introducing instrumental variables (Gage et al., 2018; Choi et al., 2020). MR analysis on general population-based datasets is a novel approach to provide evidence on causation. Our MR study based on the UK Biobank, Nurses’ Health Study, Health Professionals Follow-up Study and Women’s Genome Health Study validated that tea consumption has a mild causal relationship with obesity.

### Limitations

There are inevitable limitations that should be notified. First, potential horizontal pleiotropy could not be comprehensively assessed even though multiple sensitivity analyses were performed. However, Cochran’s Q statistic, *I* ^*2*^ statistics, MR-Egger intercept test, and MR-PRESSO global test found that there were no heterogeneity or pleiotropy in this MR analysis. Second, our study only checked the SNPs that were reported in the GWAS; the unpublished SNPs that are potentially associated with the confounders (i.e., energy intake and expenditure, exercise, physical activity, and sleep duration) might bias our findings. Third, we did not carry out subgroup analysis due to the lack of demographic information in detail. In the end, this study was on the basis of a European database. The differences in habit of tea drinking between Europeans and other races, as well as the variance within European populations living in different countries, may limit the generalizability of our results.

## Conclusion

Our findings evidenced that tea consumption has a mild causal relationship with obesity in general population. More studies are needed to clarify the effects of tea and its components on obesity-related health problems.

## Data Availability

The original contributions presented in the study are included in the article/Supplementary Material, further inquiries can be directed to the corresponding authors.
