# Supplementary material for: A Mild Causal Relationship Between Tea Consumption and Obesity in General Population: A Two-Sample Mendelian Randomization Study
Source: Front Genet. 2022 Feb 24;13:795049. doi: 10.3389/fgene.2022.795049 (PMC8907656; doi:10.3389/fgene.2022.795049)
Supplement: Supplementary file 1 [file Table1.DOCX]

Table 1 Two-sample Mendelian Randomization for tea consumption on obesity risk

| Method | N SNPs | Beta coefficient | SE | OR (95%CI) | *P* |
| --- | --- | --- | --- | --- | --- |
| IVW | 91 | -0.002 | 0.001 | 0.998 (0.996-1.000) | 0.049 |
| MR-Egger | 91 | 0.003 | 0.003 | 1.003 (0.998-1.008) | 0.255 |
| WME | 91 | -0.002 | 0.001 | 0.998 (0.996-1.001) | 0.262 |
| Weighted mode | 91 | -0.001 | 0.002 | 0.999 (0.994-1.003) | 0.505 |
| Simple mode | 91 | -0.001 | 0.003 | 0.999 (0.993-1.005) | 0.747 |

N SNPs, the number of single nucleotide polymorphisms; SE, standard error; OR, odds ratio; CI, confidence interval; IVW, inverse variance weighted; WME, weighted median estimator.
