# Supplementary material for: A Mild Causal Relationship Between Tea Consumption and Obesity in General Population: A Two-Sample Mendelian Randomization Study
Source: Front Genet. 2022 Feb 24;13:795049. doi: 10.3389/fgene.2022.795049 (PMC8907656; doi:10.3389/fgene.2022.795049)
Supplement: Supplementary file 2 [file Table2.DOCX]

[Supplementary]

Table S1 Demographics of study participants associated with tea consumption.

|  | UK Biobank 24 h recall data (n = 85,852) | | Nurses’ Health Study (n = 10,675) | Health Professionals Follow-Up Study (n = 6,618) | Women’s Genome Health Study (n = 22,691) |
| --- | --- | --- | --- | --- | --- |
| Age, years | 56.5 (7.7) |  | 52.5 (6.7) | 55.2 (8.7) | 54.7 (7.1) |
| Women | 47,786 (55.7%) | | 10,675 (100%) | 0 (0%) | 22,691 (100%) |
| BMI, kg/m^2^ | 26.7 (4.6) |  | 25.5 (4.9) | 25.8 (3.2) | 25.3 (6.7) |

BMI, body mass index; Values are n (%) or mean (standard deviation [SD]).

Table S2 Characteristics of SNPs used in the MR analysis in the summary statistics reported in the GWAS on tea consumption and obesity.

| SNP | Gene | CHR | EA | OA | SNP-exposure (tea consumption) | | | SNP-outcome (obesity risk) | | | F statistics |
| --- | --- | --- | --- | --- | --- | --- | --- | --- | --- | --- | --- |
|  |  |  |  |  | BETA | SE | P | BETA | SE | P |  |
| rs12410656 | SLC9A1 | 1 | T | C | 1.75E-02 | 3.30E-03 | 1.17E-07 | -2.86E-04 | 1.60E-04 | 7.49E-02 | 15.07 |
| rs12127737 | ARTN | 1 | A | G | 9.47E-03 | 2.05E-03 | 3.80E-06 | -2.86E-04 | 1.60E-04 | 7.49E-02 | 11.46 |
| rs143958513 | TRABD2B | 1 | T | C | 3.65E-02 | 7.55E-03 | 1.33E-06 | -2.84E-04 | 3.70E-04 | 4.42E-01 | 13.37 |
| rs35596618 | ELAVL4 | 1 | G | A | 1.46E-02 | 2.99E-03 | 9.91E-07 | 9.50E-05 | 1.46E-04 | 5.15E-01 | 12.84 |
| rs115204443 | ALG6 | 1 | G | A | 3.06E-02 | 6.58E-03 | 3.40E-06 | 6.73E-04 | 3.22E-04 | 3.65E-02 | 12.53 |
| rs290697 | NEGR1 | 1 | G | T | 8.26E-03 | 1.77E-03 | 2.97E-06 | 8.37E-05 | 8.61E-05 | 3.31E-01 | 11.71 |
| rs6593661 | LOC100996635 | 1 | A | C | 1.16E-02 | 2.49E-03 | 3.25E-06 | -1.23E-05 | 1.21E-04 | 9.19E-01 | 11.64 |
| rs10305664 | ARNT | 1 | A | C | 1.00E-02 | 1.79E-03 | 2.23E-08 | -9.69E-06 | 8.71E-05 | 9.11E-01 | 16.92 |
| rs2335252 | KCNN3 | 1 | T | G | 9.81E-03 | 2.13E-03 | 4.24E-06 | 9.85E-05 | 1.04E-04 | 3.42E-01 | 11.52 |
| rs10918630 | NOS1AP | 4 | C | T | 8.46E-03 | 1.84E-03 | 4.15E-06 | -1.41E-05 | 8.94E-05 | 8.74E-01 | 11.44 |
| rs12028567 | PAPPA2 | 1 | C | T | 8.78E-03 | 1.72E-03 | 3.53E-07 | -3.03E-05 | 8.39E-05 | 7.18E-01 | 20.84 |
| rs2813703 | ESRRG | 1 | G | A | 1.05E-02 | 1.72E-03 | 1.22E-09 | -8.30E-05 | 8.38E-05 | 3.22E-01 | 13.91 |
| rs3939638 | OR2W3 | 1 | C | T | 1.18E-02 | 2.30E-03 | 2.74E-07 | 7.71E-05 | 1.12E-04 | 4.90E-01 | 19.81 |
| rs2940806 | FOSL2 | 2 | C | T | 7.89E-03 | 1.72E-03 | 4.40E-06 | -1.03E-04 | 8.37E-05 | 2.18E-01 | 14.31 |
| rs114278367 | MSH6 | 2 | G | A | 2.79E-02 | 5.52E-03 | 4.04E-07 | 3.56E-04 | 2.70E-04 | 1.87E-01 | 11.44 |
| rs1156588 | FANCL | 2 | G | A | 1.15E-02 | 2.09E-03 | 4.00E-08 | -1.56E-05 | 1.02E-04 | 8.78E-01 | 13.00 |
| rs28691221 | GYPC | 2 | C | T | 8.55E-03 | 1.74E-03 | 9.47E-07 | -2.33E-04 | 8.55E-05 | 6.51E-03 | 14.15 |
| rs1973243 | SETD5 | 3 | T | C | 1.05E-02 | 2.19E-03 | 1.45E-06 | 1.45E-04 | 1.06E-04 | 1.74E-01 | 16.21 |
| rs2271961 | TRAIP | 3 | T | C | 1.02E-02 | 1.70E-03 | 2.03E-09 | -2.58E-05 | 8.28E-05 | 7.55E-01 | 12.98 |
| rs11715828 | FOXP1 | 3 | C | T | 7.86E-03 | 1.71E-03 | 4.23E-06 | -1.37E-04 | 8.31E-05 | 9.88E-02 | 12.94 |
| rs2117137 | EPHA3 | 3 | A | G | 1.16E-02 | 1.73E-03 | 1.92E-11 | 1.15E-05 | 8.43E-05 | 8.92E-01 | 12.52 |
| rs2937676 | LINC00901 | 3 | G | A | 8.70E-03 | 1.87E-03 | 3.37E-06 | 1.46E-05 | 9.10E-05 | 8.72E-01 | 12.86 |
| rs9866956 | OTOL1 | 3 | G | A | 9.61E-03 | 2.00E-03 | 1.56E-06 | 7.25E-06 | 9.74E-05 | 9.41E-01 | 19.27 |
| rs62296072 | FGFRL1 | 4 | T | C | 1.63E-02 | 3.41E-03 | 1.84E-06 | -4.93E-05 | 1.67E-04 | 7.67E-01 | 11.40 |
| rs1481012 | ABCG2 | 4 | G | A | 1.54E-02 | 2.70E-03 | 1.08E-08 | -7.08E-06 | 1.31E-04 | 9.57E-01 | 13.56 |
| rs4862411 | CENPU | 4 | T | C | 9.30E-03 | 2.01E-03 | 3.57E-06 | 8.45E-05 | 9.78E-05 | 3.88E-01 | 24.12 |
| rs2234233 | TAS2R1 | 5 | G | A | 1.15E-02 | 2.28E-03 | 4.80E-07 | 3.27E-05 | 1.11E-04 | 7.67E-01 | 11.68 |
| rs12518404 | NDUFAF2 | 5 | T | C | 8.13E-03 | 1.72E-03 | 2.22E-06 | -1.96E-05 | 8.36E-05 | 8.14E-01 | 12.49 |
| rs2403304 | RAB9BP1 | 5 | C | T | 8.57E-03 | 1.76E-03 | 1.16E-06 | 2.12E-05 | 8.58E-05 | 8.04E-01 | 12.44 |
| rs1368357 | MAN2A1 | 5 | C | T | 8.31E-03 | 1.79E-03 | 3.55E-06 | 1.51E-04 | 8.73E-05 | 8.29E-02 | 13.03 |
| rs7704676 | FGF1 | 5 | A | G | 1.95E-02 | 3.84E-03 | 3.71E-07 | -4.27E-05 | 1.87E-04 | 8.19E-01 | 17.58 |
| rs6872796 | LINC01470 | 5 | C | A | 1.20E-02 | 1.91E-03 | 3.36E-10 | -9.67E-05 | 9.31E-05 | 2.99E-01 | 11.53 |
| rs253602 | SGCD | 5 | T | G | 8.15E-03 | 1.72E-03 | 2.20E-06 | -4.45E-05 | 8.37E-05 | 5.95E-01 | 13.55 |
| rs11752836 | TMEM170B | 6 | A | G | 7.94E-03 | 1.71E-03 | 3.58E-06 | -9.20E-05 | 8.34E-05 | 2.70E-01 | 12.00 |
| rs144767533 | CUL9 | 6 | C | T | 1.37E-02 | 2.68E-03 | 3.46E-07 | -5.72E-05 | 1.30E-04 | 6.61E-01 | 12.68 |
| rs2504716 | PKHD1 | 6 | C | T | 1.69E-02 | 2.10E-03 | 6.63E-16 | -1.87E-04 | 1.02E-04 | 6.81E-02 | 11.58 |
| rs532243756 | LOC100507557 | 6 | T | C | 2.74E-02 | 5.46E-03 | 5.34E-07 | 1.58E-04 | 2.65E-04 | 5.52E-01 | 15.27 |
| rs9480167 | NOX3 | 6 | T | C | 1.05E-02 | 2.07E-03 | 4.69E-07 | 3.99E-05 | 1.01E-04 | 6.92E-01 | 21.79 |
| rs10238743 | SDK1 | 7 | T | G | 1.44E-02 | 3.00E-03 | 1.60E-06 | -4.19E-06 | 1.46E-04 | 9.77E-01 | 12.05 |
| rs4410790 | AHR | 7 | T | C | 2.25E-02 | 1.77E-03 | 4.22E-37 | -3.63E-05 | 8.61E-05 | 6.73E-01 | 11.59 |
| rs11768350 | LOC101927630 | 7 | C | T | 1.28E-02 | 2.34E-03 | 4.09E-08 | -3.03E-04 | 1.14E-04 | 7.67E-03 | 13.97 |
| rs7778305 | SNX13 | 7 | C | T | 8.78E-03 | 1.71E-03 | 2.72E-07 | 4.62E-05 | 8.31E-05 | 5.78E-01 | 35.12 |
| rs7799744 | DNAH11 | 7 | C | T | 1.42E-02 | 3.05E-03 | 3.25E-06 | -5.92E-05 | 1.48E-04 | 6.90E-01 | 12.26 |
| rs56163935 | SRRM3 | 7 | T | C | 1.46E-02 | 2.21E-03 | 4.59E-11 | 3.57E-05 | 1.08E-04 | 7.40E-01 | 14.04 |
| rs12538098 | MTPN | 7 | C | T | 3.69E-02 | 7.77E-03 | 2.12E-06 | 4.12E-05 | 3.78E-04 | 9.13E-01 | 14.28 |
| rs13276110 | CSMD1 | 8 | A | G | 1.02E-02 | 2.04E-03 | 5.25E-07 | -1.72E-05 | 9.94E-05 | 8.63E-01 | 13.64 |
| rs17315220 | LINC01288 | 8 | C | T | 1.13E-02 | 2.38E-03 | 1.87E-06 | 7.63E-05 | 1.16E-04 | 5.10E-01 | 12.28 |
| rs16930628 | LINC01289 | 8 | G | A | 1.17E-02 | 2.39E-03 | 9.13E-07 | 2.11E-05 | 1.16E-04 | 8.56E-01 | 14.61 |
| rs349356 | KCNB2 | 8 | G | T | 9.69E-03 | 1.87E-03 | 2.23E-07 | -8.80E-06 | 9.09E-05 | 9.23E-01 | 86.98 |
| rs10964538 | MLLT3 | 9 | T | C | 1.07E-02 | 2.27E-03 | 2.80E-06 | 2.81E-05 | 1.11E-04 | 8.00E-01 | 16.32 |
| rs77123275 | MIR6130 | 9 | C | T | 1.79E-02 | 3.87E-03 | 3.48E-06 | 1.98E-04 | 1.88E-04 | 2.93E-01 | 14.24 |
| rs11141683 | LOC494127 | 9 | A | G | 8.76E-03 | 1.81E-03 | 1.30E-06 | -5.41E-05 | 8.80E-05 | 5.39E-01 | 11.67 |
| rs12554801 | ZNF618 | 9 | C | T | 9.02E-03 | 1.81E-03 | 6.09E-07 | -4.34E-05 | 8.79E-05 | 6.21E-01 | 16.23 |
| rs12347368 | DENND1A | 9 | T | C | 1.62E-02 | 3.29E-03 | 9.27E-07 | 1.01E-03 | 5.84E-04 | 8.26E-02 | 23.32 |
| rs10906187 | CAMK1D | 10 | A | G | 9.52E-03 | 1.76E-03 | 6.35E-08 | -5.81E-05 | 8.56E-05 | 4.98E-01 | 12.56 |
| rs72802733 | SVIL-AS1 | 10 | T | C | 1.14E-02 | 2.48E-03 | 4.55E-06 | 1.85E-05 | 1.20E-04 | 8.78E-01 | 19.89 |
| rs2664404 | GRID1-AS1 | 10 | G | A | 9.22E-03 | 1.72E-03 | 8.94E-08 | 1.03E-04 | 8.39E-05 | 2.20E-01 | 13.64 |
| rs7071247 | NEURL1-AS1 | 10 | G | T | 1.25E-02 | 2.63E-03 | 2.02E-06 | 1.01E-04 | 1.28E-04 | 4.29E-01 | 12.20 |
| rs1421488 | LOC101927549 | 10 | G | A | 8.51E-03 | 1.76E-03 | 1.29E-06 | -2.33E-04 | 8.55E-05 | 6.51E-03 | 13.01 |
| rs12256016 | MGMT | 10 | A | G | 1.03E-02 | 1.95E-03 | 1.41E-07 | -1.23E-04 | 9.49E-05 | 1.97E-01 | 14.48 |
| rs17245213 | FAM99A | 11 | A | G | 1.05E-02 | 2.10E-03 | 6.33E-07 | -1.21E-04 | 1.02E-04 | 2.36E-01 | 11.79 |
| rs10832006 | ARNTL | 11 | A | G | 9.07E-03 | 1.96E-03 | 3.66E-06 | -1.12E-04 | 9.53E-05 | 2.39E-01 | 11.78 |
| rs10741694 | SOX6 | 11 | T | C | 1.04E-02 | 1.76E-03 | 4.15E-09 | 4.89E-05 | 8.58E-05 | 5.69E-01 | 12.64 |
| rs2422370 | C11orf74 | 11 | A | G | 1.17E-02 | 2.43E-03 | 1.44E-06 | -3.87E-05 | 1.18E-04 | 7.44E-01 | 13.46 |
| rs603826 | DPAGT1 | 11 | A | C | 8.94E-03 | 1.76E-03 | 4.04E-07 | 5.09E-05 | 8.58E-05 | 5.53E-01 | 12.92 |
| rs2659616 | OPCML | 11 | T | C | 7.96E-03 | 1.71E-03 | 3.12E-06 | -3.26E-05 | 8.31E-05 | 6.94E-01 | 15.75 |
| rs319277 | TAS2R42 | 12 | G | A | 1.74E-02 | 2.29E-03 | 3.71E-14 | 4.17E-05 | 1.12E-04 | 7.09E-01 | 11.31 |
| rs16914906 | PLEKHA5 | 12 | T | G | 2.98E-02 | 5.79E-03 | 2.62E-07 | 1.98E-04 | 2.81E-04 | 4.80E-01 | 15.43 |
| rs10842780 | ITPR2 | 12 | T | C | 1.11E-02 | 2.43E-03 | 4.84E-06 | 8.85E-05 | 1.18E-04 | 4.55E-01 | 12.10 |
| rs10784970 | TRHDE | 12 | C | A | 9.76E-03 | 1.99E-03 | 9.73E-07 | -1.83E-05 | 9.69E-05 | 8.50E-01 | 12.64 |
| rs10850705 | FAM222A-AS1 | 12 | C | T | 8.89E-03 | 1.73E-03 | 2.66E-07 | -1.76E-04 | 8.41E-05 | 3.64E-02 | 15.17 |
| rs9551284 | WASF3 | 13 | G | A | 8.77E-03 | 1.83E-03 | 1.57E-06 | -4.24E-05 | 8.89E-05 | 6.34E-01 | 13.32 |
| rs1577146 | LINC00433 | 13 | C | T | 8.86E-03 | 1.72E-03 | 2.50E-07 | -6.68E-05 | 8.36E-05 | 4.24E-01 | 11.55 |
| rs4468472 | ANKRD10 | 13 | T | C | 8.25E-03 | 1.74E-03 | 2.22E-06 | -2.28E-08 | 8.48E-05 | 1.00E+00 | 18.59 |
| rs34178700 | RPL10L | 14 | T | C | 8.94E-03 | 1.83E-03 | 1.03E-06 | -9.36E-05 | 8.91E-05 | 2.94E-01 | 14.89 |
| rs7161654 | SAMD4A | 14 | C | T | 9.60E-03 | 1.87E-03 | 2.81E-07 | -4.28E-05 | 9.10E-05 | 6.38E-01 | 17.47 |
| rs34940743 | NRXN3 | 14 | A | G | 9.32E-03 | 1.78E-03 | 1.73E-07 | -6.75E-05 | 8.69E-05 | 4.37E-01 | 13.79 |
| rs4363794 | GTF2A1 | 14 | A | C | 8.04E-03 | 1.71E-03 | 2.70E-06 | -8.28E-05 | 8.34E-05 | 3.21E-01 | 11.72 |
| rs17762577 | LINC00618 | 14 | C | T | 8.13E-03 | 1.71E-03 | 1.93E-06 | 1.49E-04 | 8.31E-05 | 7.37E-02 | 30.76 |
| rs12591786 | RORA-AS1 | 15 | T | C | 1.29E-02 | 2.37E-03 | 4.96E-08 | -9.16E-06 | 1.15E-04 | 9.37E-01 | 14.15 |
| rs6494791 | PAQR5 | 15 | A | G | 8.69E-03 | 1.79E-03 | 1.15E-06 | -4.92E-05 | 8.70E-05 | 5.71E-01 | 11.23 |
| rs2472297 | CYP1A1 | 15 | C | T | 3.11E-02 | 1.92E-03 | 5.20E-59 | -8.03E-05 | 9.36E-05 | 3.91E-01 | 12.91 |
| rs76207439 | MIR1538 | 16 | G | A | 1.43E-02 | 3.03E-03 | 2.17E-06 | -1.52E-04 | 1.47E-04 | 3.00E-01 | 14.44 |
| rs12446615 | LOC101928035 | 16 | G | A | 8.67E-03 | 1.71E-03 | 4.07E-07 | -2.06E-04 | 8.33E-05 | 1.37E-02 | 12.49 |
| rs2279844 | PLEKHH3, TUBG2 | 17 | A | G | 8.56E-03 | 1.76E-03 | 1.11E-06 | -8.05E-05 | 8.55E-05 | 3.47E-01 | 15.50 |
| rs73487020 | TCF4 | 18 | T | G | 1.37E-02 | 2.74E-03 | 6.01E-07 | 9.98E-06 | 1.33E-04 | 9.40E-01 | 14.30 |
| rs57631352 | STAP2 | 19 | G | A | 8.75E-03 | 1.86E-03 | 2.68E-06 | -9.56E-05 | 9.07E-05 | 2.92E-01 | 12.13 |
| rs6070328 | MIR4532 | 20 | C | T | 8.43E-03 | 1.84E-03 | 4.62E-06 | 1.38E-05 | 8.95E-05 | 8.77E-01 | 12.94 |
| rs4817505 | OLIG2 | 21 | T | C | 1.30E-02 | 1.75E-03 | 1.23E-13 | 8.10E-05 | 8.50E-05 | 3.41E-01 | 14.11 |
| rs9624470 | SPECC1L-ADORA2A | 22 | G | A | 1.52E-02 | 1.73E-03 | 1.56E-18 | 9.58E-06 | 8.43E-05 | 9.10E-01 | 14.58 |
| rs73169830 | ADORA2A-AS1 | 22 | T | C | 2.34E-02 | 3.22E-03 | 3.99E-13 | 2.30E-04 | 1.57E-04 | 1.43E-01 | 11.82 |

SNP, single nucleotide polymorphism; CHR, chromosome; EA, effect allele; OA, other allele; SE, standard error.


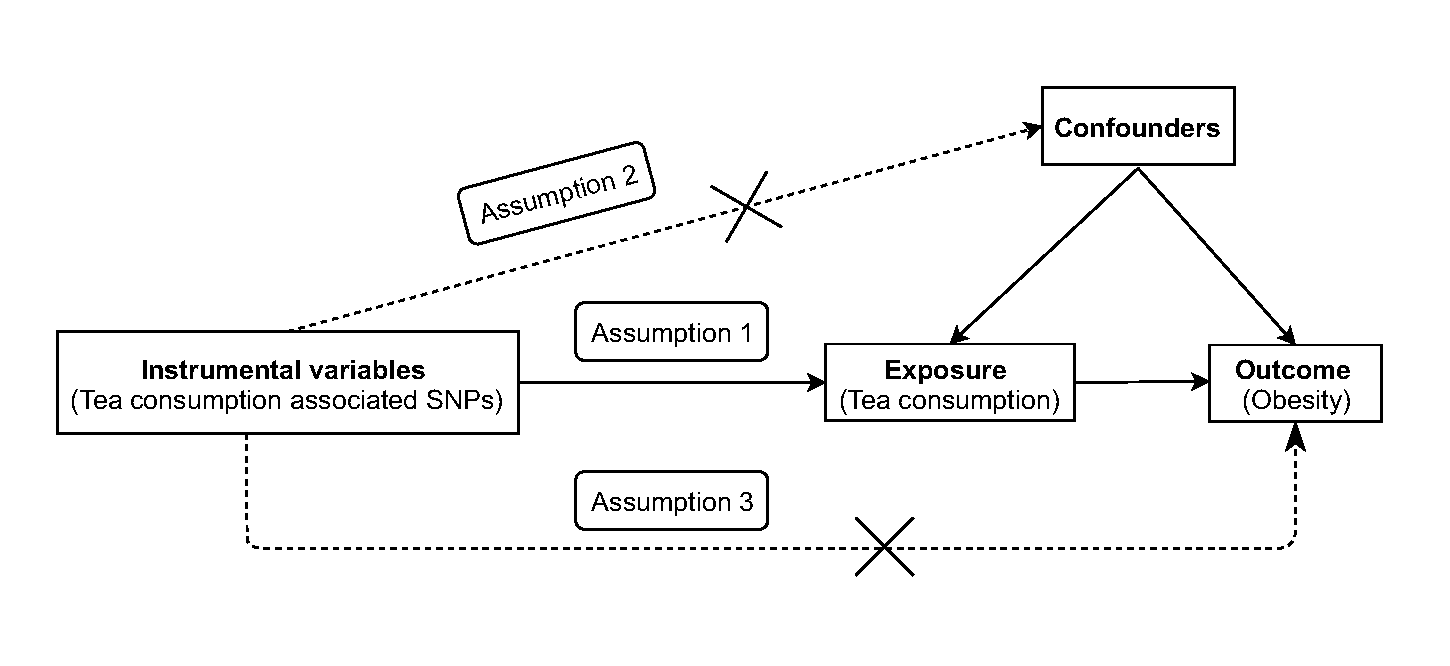


Figure S1 Diagram of the hypothesis of instrumental variables in Mendelian randomization study. SNP, single nucleotide polymorphism.
